# A Quantitative Trait Locus on Chromosome 5p Influences D-Dimer Levels in the San Antonio Family Heart Study

**DOI:** 10.1155/2010/490241

**Published:** 2010-06-27

**Authors:** V. P. Diego, L. Almasy, D. L. Rainwater, M. C. Mahaney, A. G. Comuzzie, S. A. Cole, R. P. Tracy, M. P. Stern, J. W. MacCluer, J. Blangero

**Affiliations:** ^1^Department of Genetics, Southwest Foundation for Biomedical Research, San Antonio, TX 78245-0549, USA; ^2^Department of Pathology, University of Vermont, Colchester, VT 05446, USA; ^3^Department of Medicine, University of Texas Health Science Center at San Antonio, San Antonio, TX 78229, USA

## Abstract

*Background*. D-dimer is associated with increasing severity of atherosclerosis and with increased risk of a cardiovascular disease (CVD). *Methods and Results*. To better understand this risk factor, we performed a genome scan on 803 (301 males and 502 females) Mexican Americans in the San Antonio Family Heart Study (SAFHS). The SAFHS is ideal for the discovery of quantitative trait loci (QTLs) influencing CVD because CVD risk factors are prevalent in Mexican Americans of San Antonio and because the study design involves large families, which is optimal for QTL discovery. D-dimer levels were normalized in our study. We found that D-dimer levels were heritable, at about 23% heritability (*P* ≈ .00001). In a linkage analysis employing 432 microsatellite markers, we found strong evidence of a QTL on chromosome 5p with a lod score of 3.32 at 21 centiMorgans (cM). We also found suggestive evidence of a QTL on chromosome 2q with a lod score of 2.33 at 207 cM. *Conclusions*. To our knowledge, the putative QTL on chromosome 5p is novel. The possible QTL on chromosome 2q is discussed in relation to a recent report of linkage of a related hemostatic factor to the same location. These results warrant further investigation.

## 1. Introduction

D-dimer arises as the breakdown product of the lysis of fibrin clots [1]. Studies have found that D-dimer levels are associated with an increased risk of coronary heart disease (CHD) [2–8], acute ischemic stroke [9–11], venous thrombosis [12], and peripheral arterial disease [13]. Another study found that D-dimer is also associated with severity of atherosclerosis, where severity was defined as the number of plaques detected by ultrasonography of the carotid artery [14]. The role of D-dimer as an index of functional decline seems not to be restricted to cardiovascular disease (CVD), however. Indeed, at least one study has found that D-dimer is associated with functional decline, as measured by four different indices of functional health and by mortality risk in the elderly [15]. 

Despite these results, the role of D-dimer in particular and of hemostatic factors (HFs) more generally in CVD pathology is still thought to be poorly defined [16]. One reason for this pessimistic view is that few studies have addressed the genetic architecture of D-dimer biology. Of the six studies that report heritability (*h* ^2^) estimates of D-dimer levels [17–22], only three reported significant heritability estimates for D-dimer levels, which were 65% in a UK sample population [18], 33% in a Danish sample population [21], and 25% in study comprised of sample populations from the UK and Denmark [22]. In a commentary on the genetics of HFs, it was argued by Harrap and Hopper that the genes indicated by the studies on the heritability of HFs available at the time “…may be bit-players in a much bigger picture that includes complex genetic control of a myriad of cardiovascular risk factors and that is clouded by gene-environment interactions” [23]. It would therefore seem desirable to establish heritability and then investigate if the heritability estimate, where significant, is underlain by a quantitative trait locus (QTL) or quantitative trait loci (QTLs). An excellent example of this approach was provided by the Spanish team of researchers and their collaborators cited just above [17]. They moved on from establishing the heritability of factor XII (FXII) (*h* ^2^ = 0.67) [17], which is important in thrombus formation, to demonstrating strong evidence of putative QTLs for FXII at chromosomes 5 and 2 [24]. Extending this approach of establishing heritability and identifying the main QTL or QTLs involved to the understanding of D-dimer biology would therefore seem both desirable and feasible. 

The work reported here is part of an ongoing research program on the genetic determinants of CVD in Mexican Americans participating in the San Antonio Family Heart Study (SAFHS) [25]. We decided to approach the question of D-dimer involvement in CVD in the SAFHS from a statistical genetic perspective to dissect the underlying genetic architecture of the D-dimer phenotype. The main goal under this approach is to identify novel functional genes potentially mediating D-dimer involvement in CVD. Toward this end, we carried out a multipoint genome scan of D-dimer levels in Mexican American families participating in the SAFHS. There are two reasons why we can expect this approach to be successful. First of all, there is a high prevalence of CVD risk factors, such as type 2 diabetes and overweight/obesity, in the Mexican American population of San Antonio, and, therefore, this population can be said to be relatively enriched for the genetic and environmental determinants of CVD [25]. The other reason is that our particular approach of focusing on large, extended families is thought to be the optimal design for genome-scan linkage analyses [25].

## 2. Methods

### 2.1. Study Participants and Blood Samples

The SAFHS population is comprised of large Mexican American extended families randomly ascertained with respect to CVD [25]. The SAFHS protocols were approved by the Institutional Review Board at the University of Texas Health Science Center at San Antonio, and all study participants provided written informed consent. We analyzed a random subclass of the larger SAFHS population, where the subclass was comprised of those individuals who had complete data for D-dimer levels and selected covariates (see below). We will refer to this subclass in the ensuing as the sample population. The pedigree relationships exhibited by the sample population are reported in Table 1. This information gives an idea of the extent of genetic information that is both available in the sample population and useful for the purpose of identifying QTLs. 

Fasting blood samples were obtained from study participants at the clinic exam and transported daily on dry ice to the Southwest Foundation for Biomedical Research (SFBR), San Antonio, Texas. Plasma and serum were isolated by low-speed centrifugation, and the buffy coat was harvested for DNA extraction.

### 2.2. Phenotypes and Covariates

Plasma samples were transported regularly on dry ice to the University of Vermont for the determination of D-dimer levels. D-dimer levels (ng/mL) were measured with an enzyme-linked immunosorbent assay (ELISA) using 2 monoclonal antibodies against nonoverlapping determinants of D-dimer [26]. D-dimer by this method has longitudinal within-individual variability comparable to serum cholesterol [27] and is stable in long-term storage at −70°C [28]. D-dimer levels were exactly normalized using the inverse Gaussian transformation. 

The covariates used in all analyses were age, sex, age^2^, age × sex, age^2^× sex, oral contraceptive use (i.e., exogenous hormone use), and menopause status. Age, sex, oral contraception, and menopause status are each known to have a significant effect on the risk of CVD in general [29, 30], and, in some cases, these associations seem related to D-dimer levels [6–8].

### 2.3. Genotypes and Linkage Analysis Methods

DNA extracted from lymphocytes was used in polymerase chain reactions (PCRs) for the amplification of individual DNA (*N* = 1339) at 432 dinucleotide repeat microsatellite loci (STRs), spaced approximately 10 centiMorgan (cM) intervals apart across the 22 autosomes, with fluorescently labeled primers from the MapPairs Human Screening set, versions 6 and 8 (Research Genetics, Huntsville, AL). PCRs were performed separately according to manufacturer specifications in Applied Biosystems 9700 thermocyclers (Applied Biosystems, Foster City, CA). The products of separate PCRs, for each individual, were pooled using the Robbins Hydra-96 Microdispenser, and a labeled size standard was added to each pool. The pooled PCR products were loaded into an ABI PRISM 377 or 3100 Genetic Analyzer for laser-based automated genotyping. The STRs and standards were detected and quantified, and genotypes were scored using the Genotyper software package (Applied Biosystems).

Mistyping analyses were performed on the preliminary genotype marker data using Simwalk II, following the recommendations of the program developers for accounting for mistyping error by (1) blanking the errant called alleles, (2) recalling them conditionally on the analysis, or (3) retyping the mistyped marker or markers as resources permit [31]. Our overall rate of blanking mistyped markers is 1.37%. These mistyping analyses allow investigators to account for Mendelian errors and spurious double recombinants, both of which can severely reduce the power of a linkage analysis if not accounted for [31]. On addressing mistyping error (by blanking, recalling, or retyping), these genotype data were then used to compute maximum likelihood estimates of allele frequencies in SOLAR [32]. Matrices of empirical estimates of identity-by-descent (IBD) allele sharing at points throughout the genome for every relative pair were computed using the Loki package, which uses Markov chain Monte Carlo methods [33]. The multipoint IBD matrices are required under our variance components modeling approach (see below). The Simwalk II and Loki programs both require chromosomal maps. We used the set of high-resolution chromosomal maps provided by the research group at deCODE genetics, Reykjavik, Iceland, which are available online as a supplemental table to the primary article [34]. For the identification and localization of QTLs, we performed variance components linkage analyses in SOLAR [32].

## 3. Results

The descriptive statistics for the untransformed D-dimer data are presented in Table 2. Descriptive statistics for the principal covariates (i.e., not including interactions) of age, sex, oral contraceptive use, and menopause status are also reported in Table 2.

The heritability of transformed D-dimer levels in the sample population is 0.22905 with a standard error of ±0.06792. This maximum likelihood estimate of heritability is significant relative to the null hypothesis of a heritability of 0 with a *P*-value *≈* .00001. 

**Figure 1:**
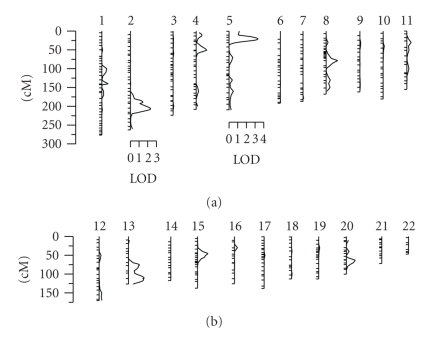
Multipoint genome-scan across the 22 autosomes for D-dimer levels in the San Antonio Family Heart Study. At all chromosomes, the vertical axis is in lod scores, and the horizontal axis is in centiMorgans (cM).

**Figure 2:**
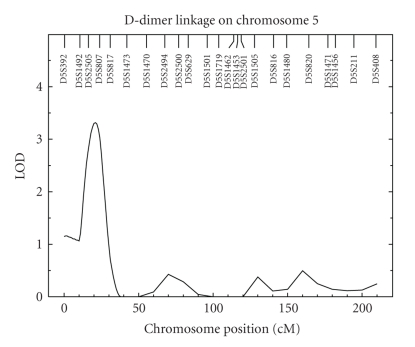
Linkage analysis results for D-dimer levels on chromosome 5. Axes are as in Figure 1.

**Figure 3:**
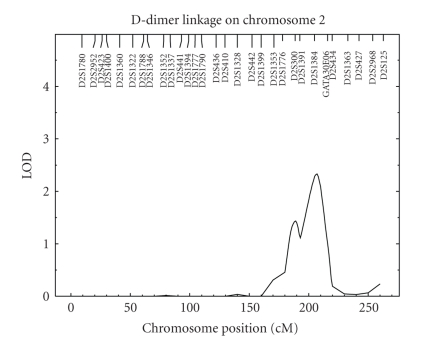
Linkage analysis results for D-dimer levels on Chromosome 2. Axes are as in Figure 1.

Encouraged by the significant heritability result for transformed D-dimer levels, we carried out a multipoint genome scan. The results of these analyses are reported in Figure 1. Our genome-wide maximum lod score is 3.32 at 21 cM on chromosome 5p15.32–p15.2 in between markers D5S2505 and D5S807 with 1-lod support interval from 14 cM to 27 cM (Figure 2). Generally, a lod score greater than 3 is deemed significant evidence for a putative QTL, and a lod score greater than 2 is taken to be suggestive evidence of a putative QTL [25]. In addition to the strong evidence of a QTL on chromosome 5p, we found suggestive evidence for another QTL on chromosome 2q33.2 with a lod score of 2.33 at 207 cM (Figure 3).

## 4. Discussion

We found that D-dimer levels are significantly heritable in the sample population of SAFHS Mexican Americans, with a heritability of about 23%. As discussed above, there are six other studies that have reported the heritability of D-dimer levels [17–22], and only three of these found a significant heritability of D-dimer levels at about 65% in a UK sample population [18], 33% in a Danish sample population [21], and 25% in study comprised of sample populations from the UK and Denmark [22]. The study by Ariëns et al. analyzed data on 118 monozygotic (MZ) and 112 dizygotic (DZ) female twins [18]. Bladbjerg et al. also analyzed twin data on D-dimer levels, and their sample size consisted of 130 MZ and 155 DZ same-sex twins [21]. Williams et al. also analyzed twin data, but this time from a mixed-population sample of 1814 UK female twins (447 DZ and 460 MZ pairs) and 398 Danish DZ twins [22]. As regards the other three, Souto et al. investigated a sample of 397 individuals from 21 extended pedigrees (12 pedigrees of which required ascertainment correction) [17], Peetz et al. examined 180 MZ and 90 DZ (same sex) twins [19], and Vossen et al. analyzed 330 individuals from a large kindred [20]. It is not clear why D-dimer levels would be heritable in some populations and not in others. One potential explanation for the discrepancy in heritability estimation across populations may be that the studies in which the heritability of D-dimer levels was not significant were relatively more youthful. The mean age in the Souto et al. study was reported as 37.7 years with a range from <1 to 88 years of age [17]. In the Peetz et al. study, the MZ twins had a mean age of 32.8 years and the same-sex DZ twins had a mean age of 35.4 years [19]. Vossen et al. reported a mean age of 31.3 years with a range from 1 to 90 years of age [20]. Ariëns et al. reported a mean age of about 53 and 52 years, respectively, for the MZ and DZ subsets of twins [18]. Bladbjerg et al. reported mean ages of 78.1 and 77.9, respectively, for the MZ and DZ subsets of twins [21]. The weighted mean age for the study by Williams et al. for mixed UK and Danish samples is a little over 53 years (calculated from their Table 1) [22]. The mean age in our study, as reported in Table 2, is about 42.5 and 43.5 years in males and females, respectively. For the male subset, the range is from 18 to 97 years of age. For the female subset, the range is from 18 to 89 years of age. It may be that both mean age and the range of ages in a study population have a significant effect in heritability determination. This is consistent with the well-known importance of age in relation to the impact of HFs on CVD risk [29]. 

We also carried out a genome-scan study of D-dimer levels in the SAFHS and found a lod score of 3.32 on chromosome 5p15.32–p15.2 at 21 cM near markers D5S2505 and D5S807 and a lod score of 2.33 on chromosome 2q33.2 at 207 cM. Our first signal is thought to be significant evidence of a putative QTL, and the second signal is thought to be suggestive evidence of another possible QTL. Incidentally, Soria et al. found evidence of QTLs at chromosomes 5q33 and 2p25, each time at the opposite end of the chromosome with respect to the current report [24]. In the only other linkage study of D-dimer levels, Williams et al. reported lod scores of 3.51 and 2.62 at chromosomes 5q14.1–q21.3 and 14q24.1–q32.13, respectively [22]. We interrogated the bioinformatics literature for any candidate genes of relevance to hemostasis within the 1-lod interval of the putative QTL on chromosome 5p15 but were unable to find any such genes. As for the putative QTL on chromosome 2q33.2, however, we found from the bioinformatics literature that the gene for tissue factor pathway inhibitor (TFPI)—TFPI is a Kunitz-type protease inhibitor that inhibits fibrin clot formation through regulation of the extrinsic pathway of coagulation—located nearby at 2q31–q32.1 is an excellent candidate gene.

Regarding the putative QTL on chromosome 5p15.32–p15.2 reported herein, we note that Sabater-Lleal et al. found a suggestive lod score of 2.31 at chromosome 5p15.32 for thrombin-activatable fibrinolysis inhibitor (TAFI), which is an inhibitor of fibrinolysis [35]. This convergence of our finding and of that by Sabater-Lleal et al. is exciting because together they suggest that the QTL potentially in common to both studies may be underlain by a novel functional gene that affects hemostasis. 

Although less strong in comparison to our main signal, the second of our above-mentioned signals is exciting to us still because a recent report of a linkage analysis of TFPI by Almasy et al. found a significant lod score of 3.52 on chromosome 2q near marker D2S1384, which is right where our signal on chromosome 2q appears and is nearby to the structural location of the TFPI gene [36]. Given that D-dimer is a degradation product of the breakdown of fibrin clots, the convergence of the signal in the work of Almasy et al. and our chromosome 2q signal is consistent with these QTLs being underlain by the TFPI gene. 

Unfortunately, we did not measure TAFI or TFPI levels. We are, however, interested in measuring these HFs and examining their potential relevance to the findings of this report. Needless to say, more investigations on the putative QTL on chromosome 5 and the possible QTL on chromosome 2 are warranted.

## Figures and Tables

**Table 1:** Pedigree relationship types in the San Antonio family heart study.

Relationship type	Number observed
Parent-Offspring	677
Full-Siblings	739
Half-Siblings	133
Grandparent-Grandchild	211
Avuncular	1543
Half-Avuncular	222
First-Cousins	1814

**Table 2:** Descriptive statistics for D-dimer levels and principal covariates.

D-dimer (ng/mL)			
Mean	Variance		*N*
105.9389	11526.4059		803

Sex	Mean age	Menopause	Oral contraceptives
Male (*N* = 301)	42.4676	NA	NA
Female (*N* = 502)	43.4676	180	78
